# Rational Drug Repurposing: Focus on Lysosomotropism, Targets in Disease Process, Drug Profile, and Pulmonary Tissue Accumulation in SARS-CoV-2 Infection/COVID-19

**DOI:** 10.3389/fphar.2020.584881

**Published:** 2020-11-20

**Authors:** Markus Blaess, Lars Kaiser, Oliver Sommerfeld, Simone Rentschler, René Csuk, Hans-Peter Deigner

**Affiliations:** ^1^ Institute of Precision Medicine, Medical and Life Sciences Faculty, Furtwangen University, Villingen-Schwenningen, Germany; ^2^ Institute of Pharmaceutical Sciences, University of Freiburg, Freiburg, Germany; ^3^ Department of Anaesthesiology and Intensive Care Medicine, Jena University Hospital, Jena, Germany; ^4^ Department of Organic Chemistry, Martin-Luther-University Halle-Wittenberg, Halle (Saale), Germany; ^5^ EXIM Department, Fraunhofer Institute IZI Leipzig, Rostock, Germany; ^6^ Faculty of Science, Tuebingen University, Tübingen, Germany

**Keywords:** SARS-CoV-2, COVID-19, lysosomotropic compounds, approved active compounds, cytokine storm syndrome, lysosomotropism, repurposing approved drugs, lysosome

## Introduction

The pandemic severe acute respiratory syndrome coronavirus 2 (SARS-CoV-2) has been identified as the disease-causing pathogen of Coronavirus disease 2019 (COVID-19). (Pre)clinical research to identify rapidly available small molecules for the treatment of SARS-CoV-2 infections/COVID-19 has focused to date on the approved lysosomotropic antimalarials chloroquine and hydroxychloroquine, the investigational remdesivir (GS-5734, compassionate use), and the anti-inflammatory corticosteroid dexamethasone (COVID-19 Treatment Guidelines Panel, 2020). Lopinavir/ritonavir and other HIV protease inhibitors, however, were discontinued as treatment options in COVID-19 demonstrating no clinical benefit in clinical trials.

Despite encouraging results in treating hospitalized patients with COVID-19 requiring supplemental oxygen, mechanical ventilation, or extracorporeal membrane oxygenation (ECMO) with remdesivir and dexamethasone, there is still a lack of active compounds exhibiting pan-coronavirus antiviral activity, tackling or preventing host cell infection, forming syncytia, endotheliitis, or the cytokine release syndrome (CRS)/cytokine storm syndrome in COVID-19. Target-oriented and in particular site of action-oriented drug repurposing of small molecules has the potential to close the gap in prophylaxis and treatment of mild and moderate COVID-19 and to reduce mortality in severe cases.

## Oxidative Stress, Apoptosis, Multinucleate Syncytia, Host Cell Entry, and Cytokine Storm Syndrome Define Drug Repurposing Targets

Oxidative stress (e.g., enhanced ROS levels) has been demonstrated in animal models of SARS (Delgado-Roche and Mesta, 2020) and serves as a possible explanation why SARS-CoV-2 patients with Glucose-6-phosphate dehydrogenase (G6PD) deficiency develop intravascular hemolysis and methemoglobinemia (Palmer et al., 2020). Both, chloroquine and hydroxychloroquine, are supposed to trigger severe drug-induced hemolytic anemia in G6PD-deficient COVID-19 patients (Beauverd et al., 2020; Kuipers et al., 2020).

Severe COVID-19 is associated with an atypical diffuse alveolar damage, ending in the acute respiratory distress syndrome (ARDS) (Huang et al., 2020), most likely accompanied by occurrence of syncytia as a result of a direct infection of cells by an infected neighboring cell without releasing a complete virus (Ou et al., 2020).

Ceramides, in particular C_18_-ceramide, are present in (sepsis-induced) cardiac dysfunction (Chung et al., 2017), and are effective in triggering exocytosis in rat PC12 cells (Tang et al., 2007); further they may contribute to SARS-CoV-2-related cell–cell fusion by exocytosis of viral S protein fractions and development of multinucleate syncytia.

Non-structural protein nsp2 of SARS-CoV-2 was associated with host cell cell cycle progression, and apoptosis in host cells, suggesting an impact on disrupting the host cell environment (Yoshimoto, 2020) and apoptosis of endothelial cells (Varga et al., 2020).

According to current knowledge, cleavage-mediated fusion of viral S protein with host cells can occur either immediately at the cell surface by TMPRSS2 or within the lysosome catalyzed by lysosomal cathepsin L (Belouzard et al., 2012). The lysosomal cathepsin L induced fusion of SARS particles bound to ACE2 with host cells (Millet and Whittaker, 2015) is sensitive to lysosomal pH. Hence both, TMPRSS2 and cathepsin L, display promising targets of prophylaxis and treatment of SARS-CoV-2 infection/COVID-19.

In severe COVID-19, SARS-CoV-2 is likely to cause both, pulmonary and systemic inflammation, thus leading to multi-organ dysfunction in high risk populations. Significantly higher concentrations of IL-8, TNFα, and IL-6 in deceased patients (Chen et al., 2020) are suggesting a rapid and severe deterioration during SARS-CoV-2 infection associated with CRS/cytokine storm syndrome (Mehta et al., 2020).

## Lysosomotropic (Active) Compounds Are Valuable Drug Candidates

**FIGURE 1:**
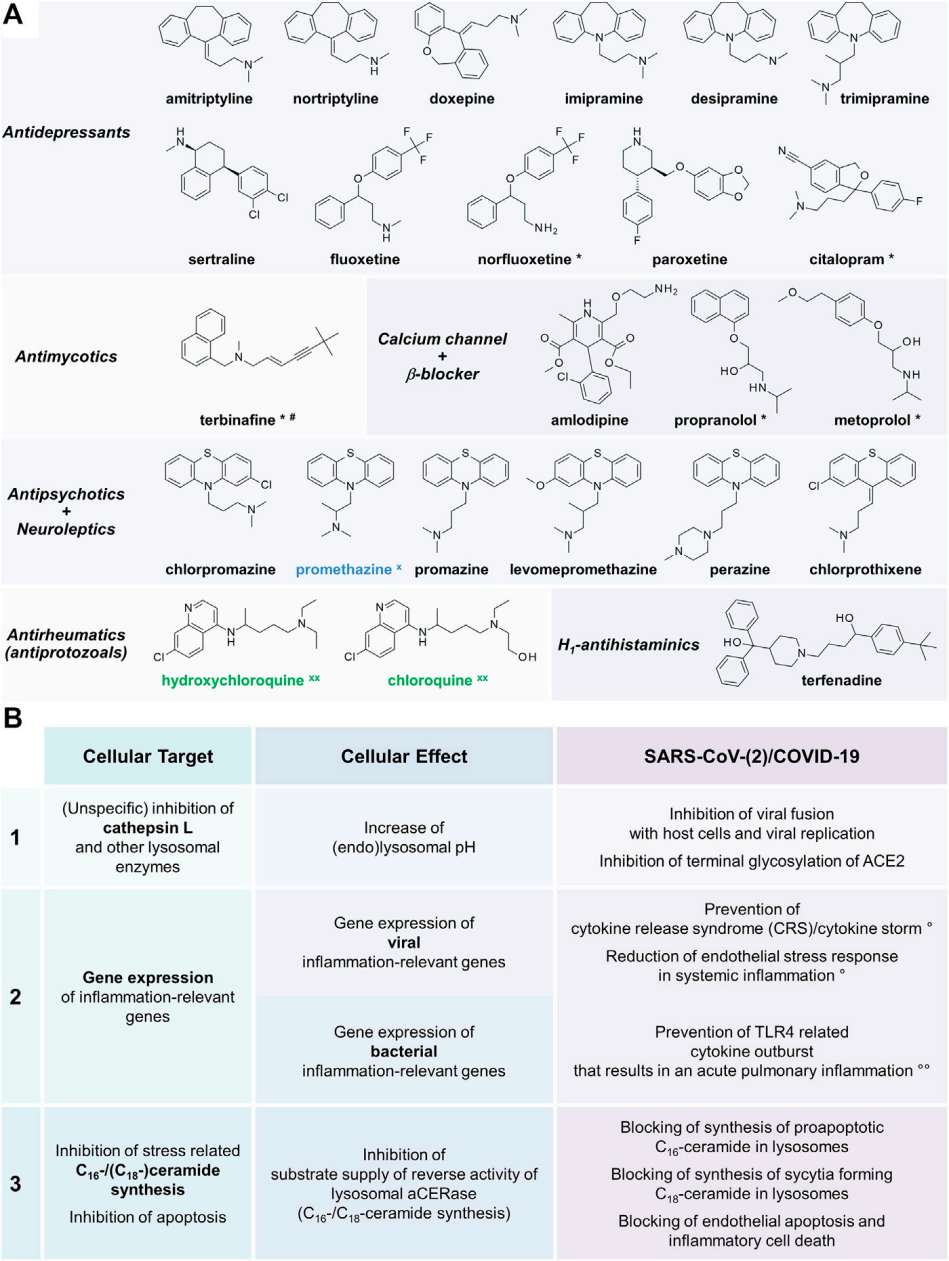
**(A)** Variety of approved lysosomotropic compounds for various indications (Kornhuber et al., 2008; Blaess et al., 2018). Achievement of the desired lysosomotropic effect depends on the active compound, the dosage, and accumulation in lysosomes. Unless indicated, maximum daily doses are split into three applications. *Lysosomotropism very likely, but not yet confirmed, lysosomal drug concentration (effect) within the therapeutic margin expected; dosage: ^#^single dose per day; ^x^ *in vitro* anti-SARS‐CoV tested, ^xx^ *in vitro* anti-SARS-CoV and anti-SARS-CoV-2 tested (Vincent et al., 2005; Kornhuber et al., 2008; Dyall et al., 2014; Zhou et al., 2016; Blaess et al., 2018; Liu et al., 2020; Weston et al., 2020). **(B)** Cellular targets, cellular effects, and effects related effects of lysosomotropic active compounds in SARS-CoV-2 infection/COVID-19 (Vincent et al., 2005; Masters, 2006; Mingo et al., 2015; Zhou et al., 2016; Blaess et al., 2018; Varga et al., 2020; Zhou et al., 2020). Lysosomotropic compounds target in mammalian cells three major targets related to SARS-CoV-2 infection/COVID-19: cathepsin L (1), gene expression of inflammation-relevant genes (2), C_16_-ceramide and C_18_-ceramide synthesis, and apoptosis of host cells (3). Addressing targets 1–3 results in various disease process interfering effects supposed to improve SARS-CoV-2 infection/COVID-19 outcome; (°) in viral infection and bacterial superinfection, (°°) only in bacterial superinfection.

Lysosomotropism is a biological characteristic of small molecules and always present in addition to intrinsic pharmacological effects. Various well-known approved drugs such as amitriptyline, chlorpromazine, sertraline, and imipramine share lysosomotropic characteristics (Figure 1A) (Kornhuber et al., 2008; Blaess et al., 2018). Regardless of their pharmacological effects, they are accumulating in lysosomes raising the lysosomal pH from 4.5–5 to 6–6.5, beyond the optimum of most of the lysosomal enzymes, including cathepsin L. Since no effects of lysosomotropic aminoglycoside antibiotics on free cathepsin L (Zhou et al., 2016) or other lysosomotropic drugs on lysosomal enzymes such as acid sphingomyelinase exist (Blaess et al., 2018), a selective inhibition is unlikely.

Lysosomotropic compounds are not limited to mediate inactivation of cathepsin L Figure 1B. Moreover, lysosomotropic compounds are assumed to suppress the CRS/cytokine storm syndrome and to attenuate the transition from mild to severe SARS-CoV-2 infection/COVID-19 (Zhou et al., 2020). Data of the lysosomotropic model compound NB 06 in LPS-induced inflammation in monocytic cells (Blaess et al., 2018) supports the hypothesis. NB 06 affects gene expression of the prominent inflammatory messengers IL1B, IL23A, CCL4, CCL20, and IL6; likewise, it has beneficial effects in (systemic) infections involving bacterial endotoxins by targeting the TLR4 receptor pathway in sepsis. Similarly, desipramine reduces endothelial stress response in systemic inflammation (Chung et al., 2017).

Apoptosis of (infected) mammalian cells is characterized by an increase in C_16_-ceramide (Thomas et al., 1999) and can be blocked via lysosomotropic compounds such as NB 06, chlorpromazine, and imipramine (Blaess et al., 2018). Furthermore, C_18_-ceramide triggered exocytosis and forming of syncytia is blocked by chlorpromazine as well (Garner et al., 2010).

## Suitable Drug Profiles and Routes of Administration

According to current knowledge, in therapy inhibition of lysosomal pH dependent processes (e.g., cathepsin L dependent viral entry into host cells) can be obtained only through off-label use of lysosomotropic drugs. Systemic application in lysosomotropic drug concentrations and obtaining an efficacious blood level is sometimes accompanied by severe adverse effects and/or (in this case) undesirable (intrinsic) pharmacological effects. Chloroquine was among the first lysosomotropic active compounds exerting antiviral effects on SARS-CoV-2 (Liu et al., 2020) and during SARS-CoV pre- and post-infection conditions (Vincent et al., 2005). Owing to an unfavorable drug profile (G6PD patients, insufficient lysosomotropism, elimination half-life of 45 ± 15 days), a recommendation against (hydroxy)chloroquine, but not against lysosomotropic active compounds in principle was issued (COVID-19 Treatment Guidelines Panel, 2020).

Chlorpromazine displayed anti-SARS-CoV-2 effects *in vitro* (Weston et al., 2020) and protective effects on COVID-19 in patients in a psychiatry hospital (NCT04366739). Consequently, chlorpromazine is rated as a promising candidate in COVID-19/CRS treatment. In case of treatment of people without mental illness, however, a premature termination of treatment due to severe side effects by systemic application of chlorpromazine is extremely likely. This raises the question of how to handle this issue to provide well tolerated lysosomotropic drugs in SARS-CoV-2 infection/COVID-19.

## Personalized Bench to Bedside Treatment Concept

Numerous available approved drugs with lysosomotropic characteristics permit tailor-made therapy. The individual pre-existing conditions are a criterion for the selection and combination of lysosomotropic drugs. For choosing suitable lysosomotropic drugs some issues have to be considered:

### Tolerable Intrinsic Pharmacology and Drug Profile

Various lysosmotropic drugs in Figure 1A demonstrated anti-SARS-CoV(-2) efficacy (Dyall et al., 2014; Zhou et al., 2016; Liu et al., 2020; Weston et al., 2020), offer a more favorable drug profile than the initially investigated chloroquine and hydroxychloroquine.

### Accumulation In Lysosomes of Pulmonary Tissue

Imipramine and chlorpromazine are accumulating in isolated perfused lung tissue and imipramine in alveolar macrophages (Wilson et al., 1982; Macintyre and Cutler, 1988) suggesting that lysosomotropic drug concentrations in pulmonary alveoli and protective effects on SARS-CoV-2 infection of particular drugs are likely. Of the lysosomotropic *in vitro* anti-SARS-CoV-2 antibiotics teicoplanin, oritavancin, dalbavancin, and telavancin (Zhou et al., 2016), solely teicoplanin and telavancin are in accumulating pulmonary tissue and are expected to be a treatment option.

### Additional Therapeutic Benefits In Sars-Cov-2 Infection/Covid-19

Beside lysosomotropism certain intrinsic pharmacological effects are advantageously in SARS-CoV-2 infection/COVID-19. The incidence of CRS/cytokine storm syndrome associated with secondary gram-positive bacterial infections is likely to be minimized by using the pulmonary tissue accumulating antibacterials teicoplanin and telavancin or the antifungal itraconazole in systemic mycoses in appropriate systemic drug levels.

### Choosing A Suitable Route of Administration

Systemic application of chlorpromazine (NCT04366739) and fluoxetine (NCT04377308) as lysosomotropic drugs may provoke severe and unfavorable adverse effects in mental healthy patients. Since the respiratory tract is both, the gateway for SARS-CoV-2 infection/COVID-19 and an internal surface, the expedient is a local application in the airways and/or the respiratory tract. Local application of small molecules is possible, preferably as inhalant or via nebulizers to avoid (undesirable) systemic effects. The majority of lysosomotropic drugs should be suitable for inhalation.

### Combination With Antivirals and Tmprss2 Inhibitors

COVID-19 originates from a SARS-CoV-2 infection that could not be tackled successfully by the immune system. The antiviral remdesivir proved to be effective in infection prophylaxis (phase 0) (de Wit et al., 2020) and viral (SARS-CoV-2) infection (phase 1) within a limited period (5–6 days), shortly after the symptoms emerge and viral shedding occurs (Mitjà and Clotet, 2020). In severe COVID-19 neither a lower mortality nor a faster clearance of viruses was observed (Wang et al., 2020). As soon as the infection initiates a CRS/cytokine storm, it is likely that the transition toward COVID-19 (phase 2), a disseminated intravascular coagulation/thrombotic microangiopathy, or a bacterial secondary infection occurs. An effective multi-drug therapy, focusing on the progression of COVID-19 and emerging severe complications, can be implemented by lysosomotropic drugs, TMPRSS2 inhibitors and antivirals.

## Nafamostat: An *In Vivo* TMPRSS2 Inhibitor?

Nafamostat is an approved protease inhibitor that inhibits TMPRSS2 (*in vitro*) (Hoffmann et al., 2020), prevents (sepsis-related) disseminated intravascular coagulation, and thrombotic microangiopathy (Okajima et al., 1995; Levi and Thachil, 2020), appears to be useful in SARS-CoV-2 infection and prophylaxis, and for patients subjected to extracorporeal circulation such as ECMO (Han et al., 2011). It is doubtful, however, whether the pulmonary concentration in therapeutically dosage (Ono Pharmaceuticals, 2020) is sufficient to generate a TMPRSS2 inhibition *in vivo* as demonstrated *in vitro* due to poor accumulation in pulmonary tissue (Midgley et al., 1994).

## Single or Multi Target Approach: Lysosomotropic Drugs vs. Antibodies

Various clinical trials are currently under way using immunomodulatory IL-1 and IL-6 inhibitors or anti-IL-6R antibodies (anakinra, tocilizumab, siltuximab, and sarilumab) in patients with COVID-19 (COVID-19 Treatment Guidelines Panel, 2020); limited data, however, is yet available. In a retrospective study using tocilizumab and hydroxychloroquine, both demonstrated a limited benefit in survival (Ip et al., 2020). Tocilizumab shortens mechanical ventilation and hospital stay in severe COVID-19 (Eimer et al., 2020), while tocilizumab is often accompanied by bacterial pneumonia 2 days after application (23%) (Pettit et al., 2020).

To improve outcome, antibody cocktails consisting of anti-IL-6, IL-1 receptor blocker, IL-1 type 1 receptor, and TNF-α are suggested (Harrison, 2020), irrespective of the risk of serious adverse effects (e.g., bacterial pneumonia) due to more pronounced interference with the immune defense. Such cocktails are intended to tackle the release of pro-inflammatory cytokines IL-1β and IL-6 mediating lung and tissue inflammation, fever, and fibrosis, as they are supposed to be responsible for the emergence of COVID-19.

Although lysosomotropic drugs likewise interfere with the immune defense, such adverse effects are not reported. In contrast to antibodies, however, only the resynthesis of IL-6 and thus the available amount is reduced, but not completely obstructed, still allowing a moderate immune response. Multitargeting on core processes of the viral infection addressing the formation of multinucleate syncytia and alteration of tissue structure, ceramide metabolism, and the release of virions could be a key advantage of lysosomotropic drugs compared to current strategies.

## Future Directions

Daunting results of (hydroxy)chloroquine in clinical trials are closely related to their drug profile and minor lysosomotropism, but not to the mode of action (lysosomotropism) in general. Observations in patients treated with chlorpromazine and the extensive accumulation of imipramine in alveolar macrophages and of both, imipramine and chlorpromazine in isolated perfused lung tissue supports the benefits of lysosomotropic drugs that are accumulating in pulmonary tissue in SARS-CoV-2 infection/COVID-19.

Promising candidates among lysosomotropic drugs in fact require more than adequate lysosomotropism; accumulation in pulmonary tissue is a prerequisite as well. It is, however, likely irrelevant whether the drug or its metabolite(s) is accumulating given the broad structural requirements for this activity. Since a large number of compounds has not yet been evaluated for lysosomotropism, many compounds beside those listed in Figure 1A are expected to meet the requirements described here and may (partially) be responsible for background immunity to SARS-CoV infection.
